# Preparation of Robust Superhydrophobic Surfaces Based on the Screen Printing Method

**DOI:** 10.3390/nano16020086

**Published:** 2026-01-08

**Authors:** Yinyu Sun, Qing Ding, Qiaoqiao Zhang, Yuting Xie, Zien Zhang, Yudie Pang, Zhongcheng Ke, Changjiang Li

**Affiliations:** School of Chemistry and Chemical Engineering, Huangshan University, Huangshan 245021, China; 106065@hsu.edu.cn (Y.S.); QDing2003@163.com (Q.D.); qqzhang200404@163.com (Q.Z.); yuting_xiei@163.com (Y.X.); zhangzienzze@163.com (Z.Z.); pangyudie@163.com (Y.P.); xiaoke1020@126.com (Z.K.)

**Keywords:** superhydrophobic surfaces, screen printing, self-cleaning, mechanical durability, chemical stability

## Abstract

The bioinspired superhydrophobic surfaces have demonstrated many fascinating performances in fields such as self-cleaning, anti-corrosion, anti-icing, energy-harvesting devices, and antibacterial coatings. However, developing a low-cost, feasible, and scalable production approach to fabricate robust superhydrophobic surfaces has remained one of the main challenges in the past decades. In this paper, we propose an uncommon method for the fabrication of a durable superhydrophobic coating on the surface of the glass slide (GS). By utilizing the screen printing method and high-temperature curing, the epoxy resin grid (ERG) coating was uniformly and densely loaded on the surface of GS (ERG@GS). Subsequently, the hydrophobic silica (H-SiO_2_) was deposited on the surface of ERG@GS by the impregnation method, thereby obtaining a superhydrophobic surface (H-SiO_2_@ERG@GS). It is demonstrated that the micro-grooves in ERG can provide a large specific surface area for the deposition of low surface energy materials, while the micro-columns can offer excellent protection for the superhydrophobic coating when it is subjected to mechanical wear. It is important to note that micro-columns, micro-grooves, and nano H-SiO_2_ jointly form the micro–nano structure, providing a uniform and robust rough structure for the superhydrophobic surface. Therefore, the combination of a micro–nano rough structure, low surface energy material, and air cushion effect endow the material with excellent durability and superhydrophobic property. The results show that H-SiO_2_@ERG@GS possesses excellent self-cleaning property, mechanical durability, and chemical stability, indicating that this preparation method of the robust superhydrophobic coating has significant practical application value.

## 1. Introduction

In nature, many plants or animals exhibit unique wetting phenomena that have attracted significant attention [1,2,3]. Among them, the bioinspired superhydrophobic material, due to its micro–nano rough structure and low surface energy chemical substances, demonstrates excellent superhydrophobic and self-cleaning properties [4,5]. At present, the methods for preparing the bioinspired superhydrophobic materials mainly include the sol–gel method [6,7], spraying method [8,9], and template method [10,11], etc. These methods aim to construct micro–nano structures similar to those of lotus leaf surfaces on different substrates such as metals [12,13], ceramics [14,15], polymers [16,17], and textiles [18,19]. Although superhydrophobic materials have shown great potential in areas such as self-cleaning [20,21], anti-icing [22,23], and anti-corrosion [24,25], they still face a series of serious challenges in the process of moving towards large-scale practical application. The most crucial aspect is that the mechanical stability of the superhydrophobic surface still needs to be further enhanced. The superhydrophobic property relies on the micro–nano structure of the material surface and low surface energy chemical substances [26,27]. However, these precise microscopic structures are very fragile and prone to damage under external mechanical actions such as friction, scraping, wear, or impact. Once the structure is damaged, air cannot be retained, and the superhydrophobic property will quickly be lost. How to design a robust surface that can maintain the superhydrophobic state and withstand mechanical stress in daily or harsh environments is the top priority of current research. To address this challenge, researchers have made various attempts to enhance the durability of superhydrophobic materials. Firstly, a polymer binder is added between the base surface and the superhydrophobic coating to enhance the adhesion between them. Zhi et al. reported that hydrophobic silica nanoparticles were stably anchored in the cured resin matrix through the surface groups of the silica nanoparticles reacting with the epoxy resin, thereby preparing a durable superhydrophobic material [28]. Huang et al. proposed that the superhydrophobic coating can be achieved by incorporating surface energy-lowering silica nanoparticles into methyl silicone resins, thereby enhancing their adhesion and increasing hardness [29]. However, the encapsulation of hydrophobic components by adhesives may lead to a decline in superhydrophobic properties, and the adhesive cannot completely cover all the hydrophobic components, resulting in the hydrophobic components on the coating surface still being prone to wear and tear. Secondly, the thicker superhydrophobic coating can be prepared to resist the loss of the superhydrophobic material during processes such as wear and impact. Zhang et al. provided a facile approach for the preparation of the electrodeposited silica coating by electrophoresis of superhydrophobic SiO_2_ nanoparticles into a matrix, which provides durable superhydrophobicity even though the thickness of the highly porous SiO_2_ film backbone is partially destroyed [30]. Zhang et al. proposed that the robust superhydrophobic elastic silicone with stable Cassie–Baxter state retains excellent liquid-repellent properties by removing the uppermost layer by sandpaper abrasion or by limited heat treatment [31]. This thicker hydrophobic coating has weak adhesion to the base material, which may cause the hydrophobic coating to fall off the surface of the material. And the lack of protective measures for the drophobic components is not an ideal solution for preparing durable superhydrophobic materials. Finally, more advanced and sophisticated lithography technique has been employed to enhance the durability of superhydrophobic materials. Wang et al. successfully fabricated micro-structured armor on the surfaces of silicon wafers, metals, and glass using techniques such as lithography and cold/hot pressing. They also filled the armor with nano-silica materials, successfully creating an ultra-hydrophobic surface with enhanced durability [32]. Lee et al. prepared a nanostructured glass surface through a multi-step colloidal lithography and etching process. After coating with perfluoropolyether, this material exhibited superhydrophobicity [33]. These high-precision lithography techniques have drawbacks such as high equipment costs, high energy consumption, and difficulties in their industrialization application. To sum up, developing a simple, rapid, low-cost, and compatible with the existing industrial system technology for large-scale production is the key bottleneck for the industrialization of robust superhydrophobic materials.

Herein, we take the glass slide (GS) as an example. The epoxy resin grid (ERG) coating was uniformly and densely anchored on the surface of GS (ERG@GS) through the screen printing method and high-temperature curing. Then, hydrophobic silicon dioxide (H-SiO_2_) was deposited on the surface of ERG@GS by the impregnation method, thereby obtaining a superhydrophobic surface coating (H-SiO_2_@ERG@GS). The samples were characterized by field emission scanning electron microscopy (FESEM), energy dispersive spectroscopy (EDS), and X-ray photoelectron spectroscopy (XPS). Furthermore, the superhydrophobic property, self-cleaning property, mechanical durability, and chemical stability of H-SiO_2_@ERG@GS were also investigated.

## 2. Materials and Methods

### 2.1. Materials

Screen mesh (500 mesh) was purchased from Taobao (Fuyang, China), which was ultrasonically cleaned in anhydrous ethanol for 1 h and dried at 60 °C for 2 h before using. Phenolic epoxy resin and curing agent for epoxy resin were obtained from Rhown Reagent Co., Ltd. (Shanghai, China). The H-SiO_2_ was obtained from Macklin biochemical technology Co., Ltd. (Shanghai, China). All reagents were not further treated before use.

### 2.2. Fabrication of ERG@GS

First, phenolic epoxy resin (3 g) and epoxy resin curing agent (1 g) were mixed at a speed of 200 r/min for 10 min to obtain a uniform epoxy resin mixture. Second, the screen mesh (500 mesh) was fixed on the surface of GS (2.5 cm × 7.5 cm), then the epoxy resin mixture was dripped onto the screen mesh, and scraped evenly with a scraper. Subsequently, the sample was placed into an oven at 120 °C for 1 h of high-temperature curing. Finally, the screen mesh was removed to prepare ERG@GS after the sample cooled to room temperature.

### 2.3. Preparation of H-SiO_2_@ERG@GS

A total of 2 g of H-SiO_2_ nanoparticles was added gradually to 20 mL of anhydrous ethanol, followed by ultrasonic dispersion for 10 min and stirring at a speed of 400 r/min for 30 min to obtain the H-SiO_2_ ethanol solution. Then, ERG@GS was immersed in the above mixture for 5 min. Finally, the sample was taken out and dried at 80 °C for 30 min in an oven to prepare the superhydrophobic H-SiO_2_@ERG@GS.

### 2.4. Characterizations

The microscopic morphology of the sample was visualized by FESEM (Zeiss Sigma 300, Oberkochen, Germany). XPS (Thermo Scientific K-Alpha, Waltham, MA, USA) and EDS (Oxford X-Max^N^ 80, Oxford, UK) were used to measure the surface chemical composition of the sample. The water static contact angle was measured using the contact angle goniometer (DSA100, KRUSS, Hamburg, Germany) at room temperature.

### 2.5. Performance Testing

The self-cleaning performance of H-SiO_2_@ERG@GS was observed by recording the surface adhesion phenomenon after the methyl blue aqueous solution was dropped onto the sample surface, and observing the process of sand grains being carried away from the sample surface as water droplets fell. In addition, in order to study on the chemical stability of H-SiO_2_@ERG@GS, the sample was subjected to a high temperature resistance test within a temperature range of 40 °C to 200 °C for 5 h, and immersed in different pH values (pH = 1 to 13) of acid/alkali solutions for 72 h to evaluate the results of the acid/alkaline resistance test. The water static contact angle of the treated sample was measured to evaluate the chemical stability. Furthermore, the mechanical stability of H-SiO_2_@ERG@GS was investigated. The sandpaper abrasion test involves placing a 100 g weight on the sample to make it come into contact with the sandpaper (Grit No. 1000 mesh), and then moving it 100 mm with the help of an external force. This process is repeated 300 times, and the water static contact angle of the treated sample was measured to evaluate the performance of the sandpaper abrasion test. For the tape peeling test, a standard pressure-sensitive tape (width 20 mm) was pressed on the sample surface. A steel cylindrical weight with a mass of 100 g was pressed onto the tape and rolled evenly for 5 times so that the tape and the sample surface were fully adhered. Then, the tape was quickly removed, and this process was repeated 100 times. A new adhesive tape was employed after each cycle. The water static contact angle of the treated sample was measured to evaluate the durability of the coated surfaces. Results are represented as mean ± standard deviation (n = 3).

## 3. Results and Discussion

**Figure 1:**
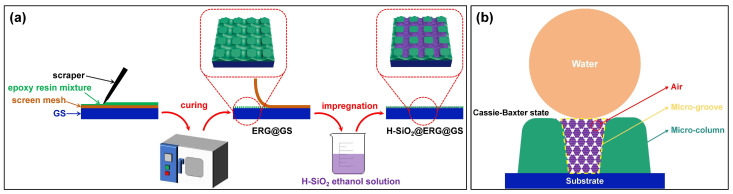
Schematic illustration of the preparation of H-SiO_2_@ERG@GS (**a**). Schematic diagram of the superhydrophobic mechanism of H-SiO_2_@ERG@GS (**b**).

Figure 1a presents the schematic diagram of the preparation of H-SiO_2_@ERG@GS by the screen printing method and the impregnation method. Firstly, the epoxy resin mixture was dripped onto the surface of the screen mesh and GS, then evenly spread with a scraper. After the sample was subjected to high-temperature curing, the screen mesh was torn off, thereby obtaining ERG@GS. In this process, the excellent fluidity and high-temperature curing property of the epoxy resin mixture effectively enhance the adhesion between the coating and the GS surface, as well as facilitating the formation of ERG. Meanwhile, the microporous structure of the screen mesh not only enables the formation of micro-grooves on the GS surface but also allows for the creation of protective micro-column structures. As shown in Figure 1b, the bottom and sidewalls of the micro-grooves provide a large specific surface area for the deposition of hydrophobic nanomaterials. Furthermore, the micro-column structure can provide excellent protection when the superhydrophobic surface is subjected to mechanical damage. Secondly, ERG@GS was put into the ethanol solution of H-SiO_2_ to form the superhydrophobic H-SiO_2_@ERG@GS. The deposition of H-SiO_2_ with low surface energy in the micro-grooves of ERG can effectively delay the escape of the air layer on the coating surface. A large amount of air is trapped between the H-SiO_2_ nanoparticles and within the epoxy resin micro-grooves, thereby significantly reducing the liquid/solid interface. This typical lotus-like micro/nano hierarchical surface structure endows the H-SiO_2_@ERG@GS with the stable Cassie–Baxter state, thereby endowing it with outstanding superhydrophobic properties. Meanwhile, the micro-grooves and micro-columns can effectively prevent H-SiO_2_ from falling off the surface of the ERG@GS or being damaged by mechanical wear, leading to endow the material with excellent durability.

**Figure 2:**
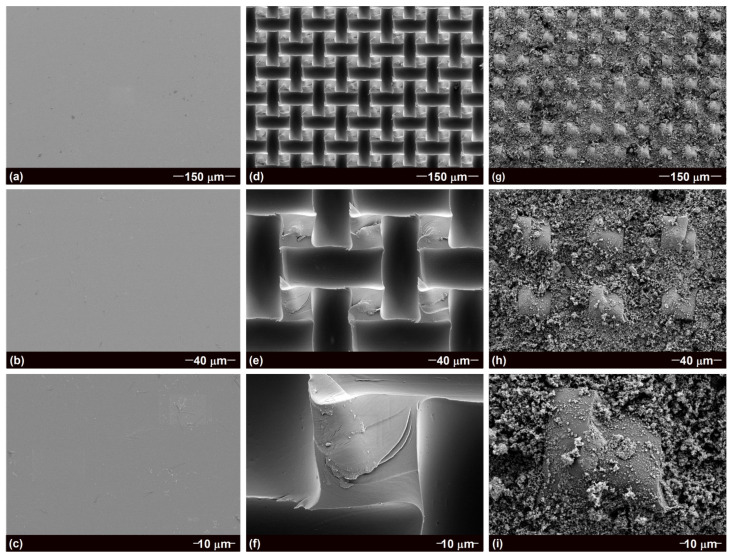
FESEM images of GS (**a**–**c**), ERG@GS (**d**–**f**), and H-SiO_2_@ERG@GS (**g**–**i**).

The surface morphology of GS, ERG@GS, and H-SiO_2_@ERG@GS was observed by FESEM. As shown in Figure 2a–c, the unprocessed GS exhibits a smooth and flat surface, which is not conducive to the direct loading of H-SiO_2_. To achieve better adhesion, it is crucial to add an epoxy resin between GS and H-SiO_2_. ERG can be uniformly and densely distributed on the surface of GS through the screen printing method and high-temperature curing (Figure 2d–f). The width of the micro-groove is found to be approximately 29 μm, which was formed by the removal of the yarns of the screen mesh. Therefore, the width of the micro-groove can be changed by selecting different diameters of the yarns of the screen mesh. The micro-grooves can provide a large specific surface area for the loading of H-SiO_2_. Meanwhile, the microporous structure of the screen mesh can form epoxy resin micro-column structures with the length and width of both approximately 24 μm, which can provide excellent protection for the superhydrophobic surface when it suffers mechanical damage. After the solution impregnation method, the dense H-SiO_2_ was loaded into the uniformly distributed micro-grooves, leading to the preparation of H-SiO_2_@ERG@GS (Figure 2g–i). It is particularly important to note that the micro-column and H-SiO_2_ deposited in the micro-grooves can effectively prevent the escape of air from the coating surface, thus further enhancing the air cushion effect. Therefore, the H-SiO_2_@ERG@GS exhibits excellent superhydrophobic properties.

**Figure 3:**
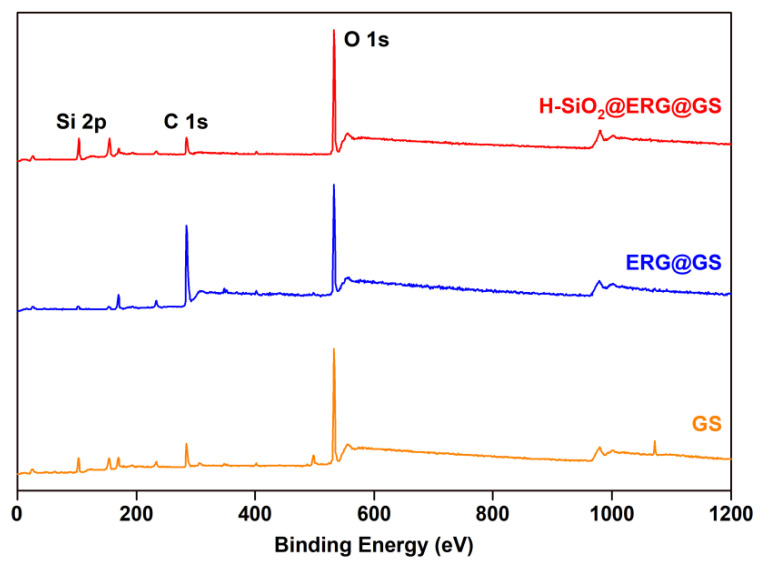
XPS analysis of GS, ERG@GS, and H-SiO_2_@ERG@GS.

**Figure 4:**
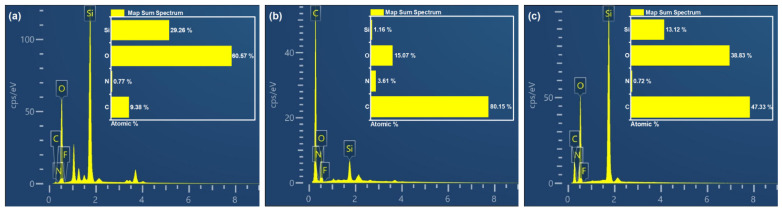
EDS spectrum of GS (**a**), ERG@GS (**b**), and H-SiO_2_@ERG@GS (**c**).

As described in Figure 3 and Figure 4, the chemical composition of GS, ERG@GS, and H-SiO_2_@ERG@GS was measured by using XPS measurements and an EDS spectrum. In Figure 3, since the main component of the GS surface is silicon dioxide, its XPS results demonstrate the existence of Si (110.6 eV) and O (545.6 eV) elements on the surface of GS, which are consistent with the EDS results (Figure 4a). As shown in Figure 3 and Figure 4b, after the loading of the epoxy resin layer on the surface of GS, the Si 2p signal peak on the surface of ERG@GS was significantly weakened, and the percentage of silicon atoms decreased from 29.26% to 1.16%. Meanwhile, the element content of C on the surface of ERG@GS has increased relatively, and the percentage of carbon atoms has also increased from 9.38% to 80.15%. The main reason for this result is that the surface of GS has been uniformly covered with the epoxy resin layer, which leads to a significant reduction in the detectable amount of silica. Compared with ERG@GS, the Si 2p signal peak on the surface of H-SiO_2_@ERG@GS surface has again intensified (Figure 3), and the percentage of silicon atoms has also increased to 13.12% (Figure 4c). This result confirms that the H-SiO_2_ has been successfully loaded on the surface of ERG@GS.

**Figure 5:**
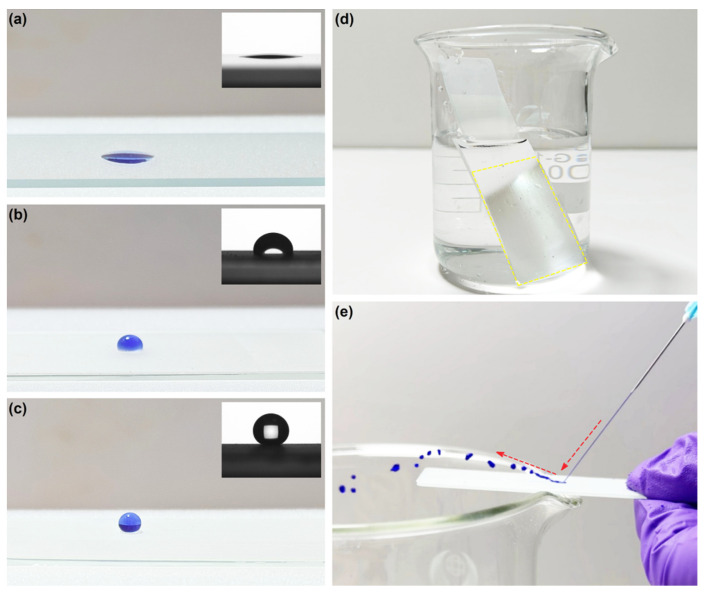
The water static contact angle and photos of water droplets on the surface of GS (**a**), ERG@GS (**b**), and H-SiO_2_@ERG@GS (**c**). The mirror-like phenomenon photo on the surface of H-SiO_2_@ERG@GS in water (**d**). The water column reflects an image on the surface of H-SiO_2_@ERG@GS (**e**).

To visually demonstrate the superhydrophobic property of the sample, photos of water droplets on the surface of GS, ERG@GS, and H-SiO_2_@ERG@GS, and the water static contact angle were observed (Figure 5a–c). As can be seen from Figure 5a, the water droplet spreads completely on the surface of GS at an angle less than 30°, thus demonstrating excellent hydrophilicity. The water static contact angle result of GS also confirms this phenomenon. After ERG was uniformly loaded on the surface of GS, the surface of ERG@GS exhibited a certain degree of hydrophobicity compared with GS, with the water static contact angle being approximately 92° (Figure 5b). As shown in Figure 5c, the water droplet keeps nearly a perfect spherical shape on the surface of H-SiO_2_@ERG@GS, indicating that it has excellent superhydrophobic properties with a water static contact angle of 158°. The above results indicate that the uniformly distributed grid-like epoxy resin layer can significantly increase the specific surface area for the large-scale loading of H-SiO_2_. Moreover, the low surface energy of H-SiO_2_ and the air cushion effect enable the modified H-SiO_2_@ERG@GS to successfully achieve superhydrophobicity. As shown in Figure 5d, the obvious mirror-like phenomenon observed on the surface of H-SiO_2_@ERG@GS in water also strongly supports the fact that the air layer on the superhydrophobic surface can create an ideal total reflection interface, thus achieving the Cassie–Baxter state. An interesting phenomenon is that the methylene blue aqueous solution was directed at the H-SiO_2_@ERG@GS surface at a certain angle using a syringe, and the water flow would reflect at a certain angle, which further demonstrated the excellent superhydrophobic property of H-SiO_2_@ERG@GS (Figure 5e). It is worth noting that the preparation method of this superhydrophobic coating has also been applied to a metal aluminum sheet and copper sheet. The results further demonstrate that this method is applicable and universal to various material surfaces, as shown in Figure S1.

**Figure 6:**
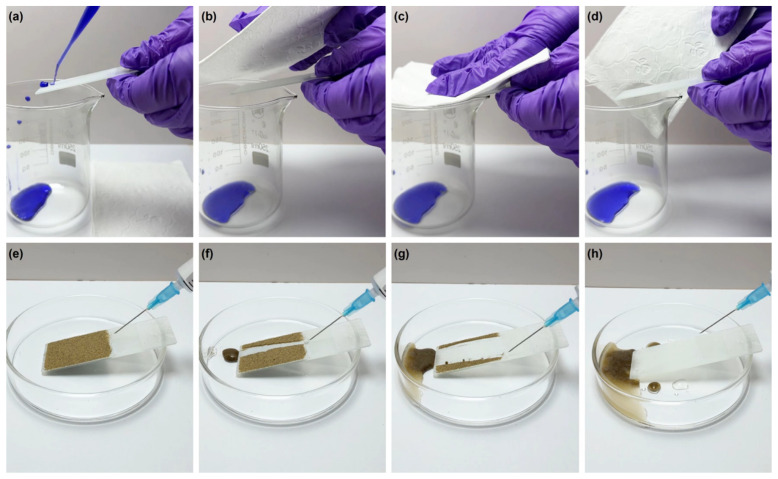
Antifouling performance of H-SiO_2_@ERG@GS testing by the methylene blue aqueous solution (**a**–**d**); Evolution processes of the self-cleaning behavior of H-SiO_2_@ERG@GS using sand grains as contaminants (**e**–**h**).

The self-cleaning and antifouling performance of H-SiO_2_@ERG@GS are among the key factors that ensure the material maintains excellent superhydrophobic properties even when it inevitably becomes contaminated by various pollutants during the actual usage process. The antifouling performance of H-SiO_2_@ERG@GS testing by the methylene blue aqueous solution and the evolution processes of the self-cleaning behavior of H-SiO_2_@ERG@GS using sand grains as contaminants are shown in Figure 6. As shown in Figure 6a–d, after the methylene blue aqueous solution was dropped onto the surface of H-SiO_2_@ERG@GS, the sample surface was wiped with a paper towel, and no obvious stains were found. This result indicates that the sample surface can prevent liquid penetration and contamination due to the presence of the low surface energy of H-SiO_2_ and the effect of the air cushion, thus possessing excellent anti-fouling ability. As can be seen from Figure 6e–h, in the self-cleaning process, the surface of H-SiO_2_@ERG@GS was contaminated with the hydrophilic sand grains. When the water droplets fall down and come into contact with the material surface, the adhesion force between the water droplets and the material surface is very weak due to the presence of low surface energy H-SiO_2_. During the rolling process, the water droplets come into contact with the hydrophilic sand grains and exert a capillary force on the sand grains. It is particularly important to note that the water droplets can remove the sand grains from the superhydrophobic surface due to the capillary force between the water droplets and the sand grains being greater than the adhesion force between the sand grains and the surface of H-SiO_2_@ERG@GS, thereby achieving a very significant self-cleaning effect.

**Figure 7:**
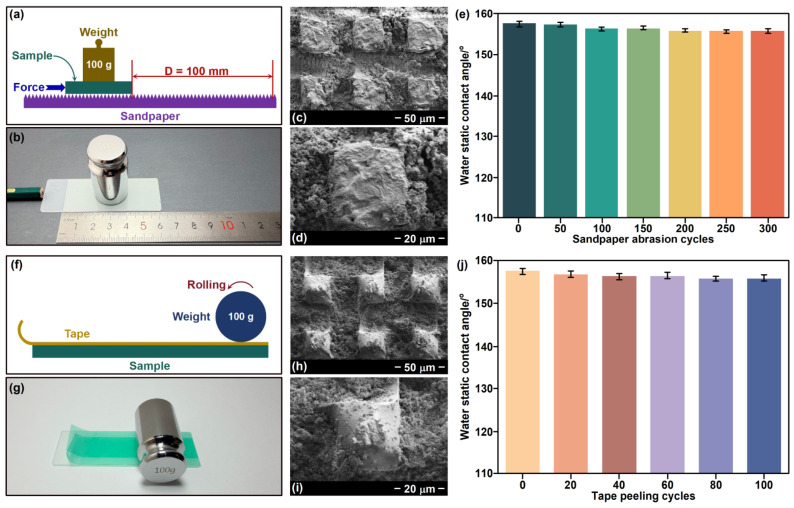
(**a**) Schematic of the sandpaper abrasion test. (**b**) A digital photograph of the sandpaper abrasion test. (**c**,**d**) FESEM images of H-SiO_2_@ERG@GS after the sandpaper abrasion test repeated 300 times. (**e**) The water static contact angle results of the sandpaper abrasion test; (**f**) Schematic of the tape peeling test. (**g**) A digital photograph of the tape peeling test. (**h**,**i**) FESEM images of H-SiO_2_@ERG@GS after the tape peeling test repeated 100 times. (**j**) The water static contact angle results of the tape peeling test. Results are represented as mean ± standard deviation (n = 3).

The mechanical durability is a crucial indicator for superhydrophobic materials in practical applications. As shown in Figure 7, the typical sandpaper abrasion test and tape peeling test were conducted. In the sandpaper abrasion test, the prepared sample was placed on a sandpaper (Grit No. 1000 mesh). Then, a steel cylindrical weight with a mass of 100 g was placed on the sample to make it come into contact with the sandpaper. Adding additional thrust enables the sample to move 100 mm along the surface of the sandpaper, and this process is repeated 300 times (Figure 7a,b). After 300 cycles of sandpaper abrasion test, the FESEM image of H-SiO_2_@ERG@GS is shown in Figure 7c,d. Clear scratches could be observed on the surface of the epoxy resin micro-columns. However, a large amount of hydrophobic H-SiO_2_ remained in the micro-grooves, which fully demonstrated the significant role of the construction of the ERG in the durability of the superhydrophobic surface. The water static contact angle results of the sandpaper abrasion test are shown in Figure 7e. After the sandpaper abrasion test, the surface of H-SiO_2_@ERG@GS still maintained excellent superhydrophobic property with its water static contact angle exceeding 150°. As can be seen from Figure 7f,g, in the tape peeling test, the adhesive tape was adhered to the surface of the sample, and then a steel cylindrical weight with a mass of 100 g was placed at one end of the tape. The weight rolled from one end to the other of the tape under the applied force, and this process was repeated five times, resulting in a complete adhesion between the tape and the sample. Subsequently, the tape was peeled off, and this process was recorded as a tape peeling cycle. This tape peeling cycle was repeated 100 times. As shown in Figure 7h,i, the micro-columns on the surface of H-SiO_2_@ERG@GS remained intact, and there were still a large amount of hydrophobic components in the micro-grooves after the tape peeling test repeated 100 times. The results indicate that the superhydrophobicity of the material surfaces is not deteriorated after 100 cycles of the tape peeling test with the water static contact angle values larger than 150°, demonstrating its good mechanical durability (Figure 7j). The main reason for those results is that H-SiO_2_ with low surface energy in the material is deposited in the micro-grooves of ERG, thus being less susceptible to wear or adhesion. Meanwhile, the raised micro-columns of ERG can provide excellent protection during the mechanical abrasion or tape adhesion process. It is particularly important to note that compared with the superhydrophobic mechanical durability tests reported in the previous literature [34,35], the sandpaper abrasion test was repeated 300 times and the tape peeling test repeated 100 times conducted on the H-SiO_2_@ERG@GS have significant advantages, which indicates that this material possesses excellent mechanical durability.

**Figure 8:**
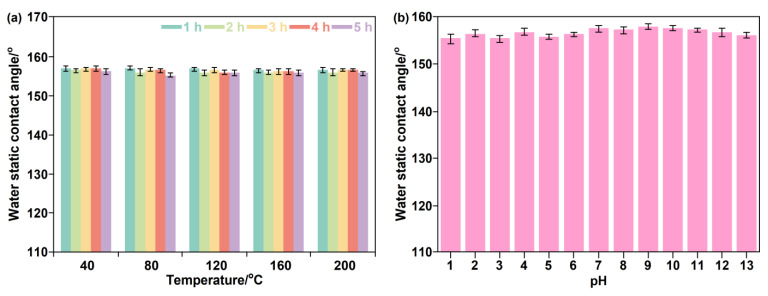
The water static contact angle results of H-SiO_2_@ERG@GS after different temperature treatment (**a**) and acid/alkaline solutions (**b**). Results are represented as mean ± standard deviation (n = 3).

The chemical stability is of great significance for the durability of superhydrophobic materials in complex practical usage environments. Therefore, the thermal stability and acid/alkaline resistance of H-SiO_2_@ERG@GS were tested. Figure 8a shows the results of the water static contact angle of H-SiO_2_@ERG@GS after being exposed to different temperature environments. As the temperature increases from 40 °C to 200 °C, the water static contact angle of the samples remains above 150°, demonstrating their excellent superhydrophobicity. The main reason is that the higher temperature does not cause the softening deformation of ERG or the collapse of the micro–nano structure, effectively protecting the surface roughness of the superhydrophobic material. Therefore, H-SiO_2_@ERG@GS has good thermal stability, which greatly expands its practical application fields, such as high-temperature industrial environments, etc. In the acid/alkaline resistance test, the samples were immersed in different pH values (pH = 1 to 13) of acid/alkaline solutions for 72 h. The water static contact angle of the samples was measured to evaluate their acid/alkaline resistance performance. As shown in Figure 8b, the water static contact angle of H-SiO_2_@ERG@GS remains exceeding 150° under corrosive conditions. The main reason is that the micro–nano structure of ERG did not dissolve or corrode under acid-base conditions because of its excellent acid/alkaline resistance, resulting in no reduction in roughness of the samples. At the same time, the micro-columns and micro-micro-grooves structure of ERG can reduce the contact between hydrophobic components and the acid/alkaline solutions, thus providing a good protective effect.

## 4. Conclusions

In summary, we have proposed a simple and efficient strategy to fabricate robust superhydrophobic materials using the screen printing method and the impregnation method. By using the screen printing method and high-temperature curing to uniformly and firmly anchor the ERG with micro-column and micro-groove structures onto the GS surface, it plays a crucial role in constructing a durable and rough surface with micro–nano structures. Meanwhile, the micro–nano rough structure and low surface energy substances are formed on the sample surface through the uniform deposition of H-SiO_2_ on the surface of ERG, resulting in a superhydrophobic surface with the water static contact angle exceeding 150°. The prepared H-SiO_2_@ERG@GS also possesses excellent self-cleaning and anti-fouling properties, mechanical durability, and chemical stability, which makes this material show great potential in practical applications. Additionally, it is particularly worth noting that this method can be applied to the surfaces of various materials (such as metals, glass, etc.), thus having a very promising application prospect.

## Data Availability

The data presented in this study are available on request from the corresponding author.

## Supplementary Materials

The following supporting information can be downloaded at: https://www.mdpi.com/article/10.3390/nano16020086/s1, Figure S1. The water static contact angle and photo of water droplets on the surface of untreated aluminum sheet (a); FESEM images of untreated aluminum sheet (b–d); The water static contact angle and photo of water droplets on the surface of coated aluminum sheet (e); FESEM images of coated aluminum sheet (f–h); The water static contact angle and photo of water droplets on the surface of untreated copper sheet (i); FESEM images of untreated copper sheet (j–l); The water static contact angle and photo of water droplets on the surface of coated copper sheet (m); FESEM images of coated copper sheet (n–p).
