# Supplementary material for: Preparation of Robust Superhydrophobic Surfaces Based on the Screen Printing Method
Source: Nanomaterials (Basel). 2026 Jan 8;16(2):86. doi: 10.3390/nano16020086 (PMC12844342; doi:10.3390/nano16020086)
Supplement: Supplementary file 1 [file nanomaterials-16-00086-s001.zip › nanomaterials-4068461-supplementary.pdf]

nanomaterials

Supplementary Materials

Preparation of Robust Superhydrophobic Surfaces Based on the Screen Printing Method

Yinyu Sun, Qing Ding, Qiaoqiao Zhang, Yuting Xie, Zien Zhang, Yudie Pang, Zhongcheng Ke, Changjiang Li *

School of Chemistry and Chemical Engineering, Huangshan University, Huangshan 245021, China; 106065@hsu.edu.cn (Y.S.); QDing2003@163.com (Q.D.); qqzhang200404@163.com (Q.Z.); yuting_xiei@163.com (Y.X.); zhangzienzze@163.com (Z.Z.); pangyudie@163.com (Y.P.); xiaoke1020@126.com (Z.K.)

* Correspondence:309003@hsu.edu.cn; Tel.: +86-13705592846

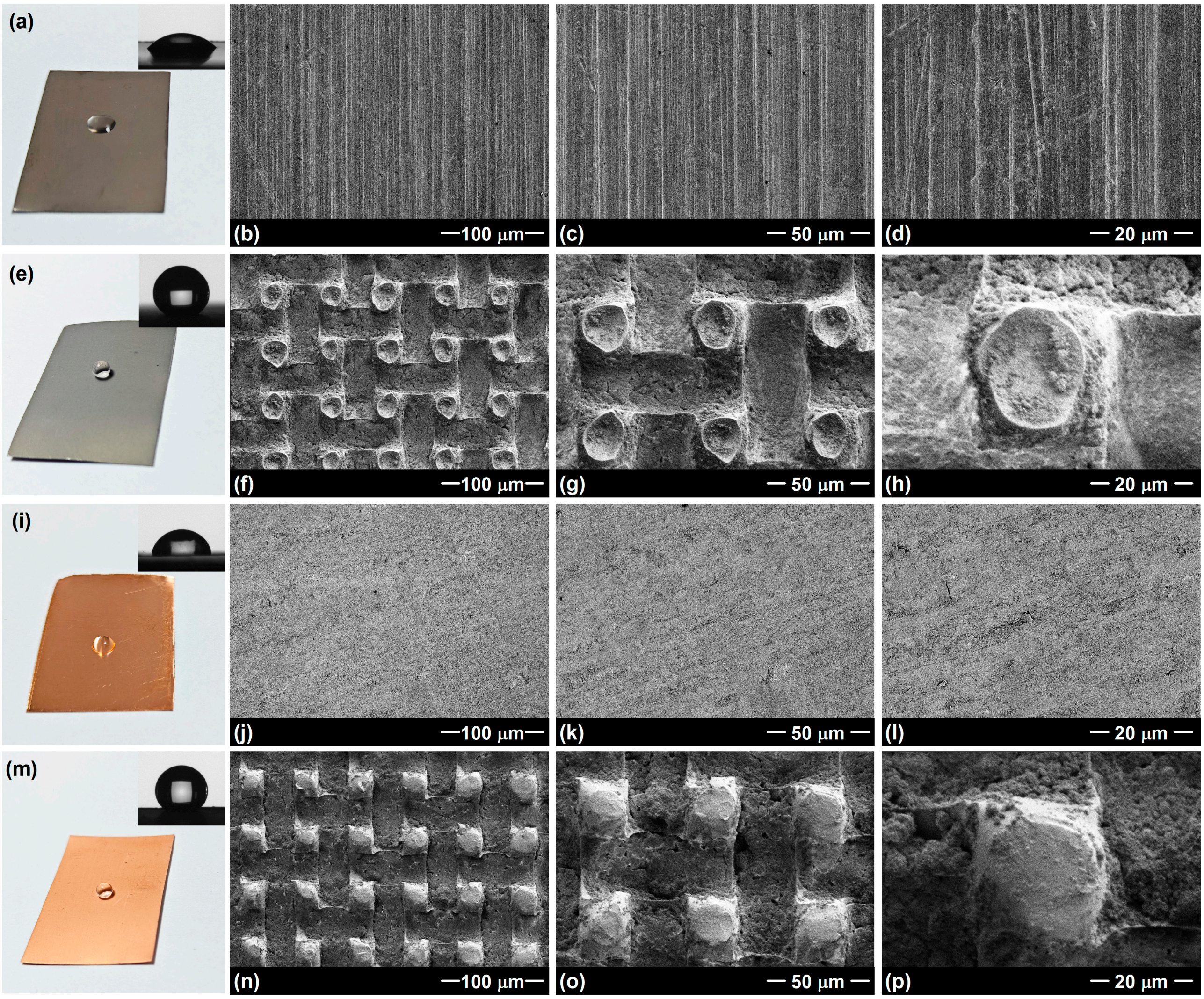

Figure S1. The water static contact angle and photo of water droplets on the surface of untreated aluminum sheet (a); FESEM images of untreated aluminum sheet (b–d); The water static contact angle and photo of water droplets on the surface of coated aluminum sheet (e); FESEM images of coated aluminum sheet (f–h); The water static contact angle and photo of water droplets on the surface of untreated copper sheet (i); FESEM images of untreated copper sheet (j–l); The water static contact angle and photo of water droplets on the surface of coated copper sheet (m); FESEM images of coated copper sheet (n–p).

 https://doi.org/10.3390/nano16020086

Disclaimer/Publisher's Note: The statements, opinions and data contained in all publications are solely those of the individual author(s) and contributor(s) and not of MDPI and/or the editor(s). MDPI and/or the editor(s) disclaim responsibility for any injury to people or property resulting from any ideas, methods, instructions or products referred to in the content.

https://doi.org/10.3390/nano16020086
